# The effect of Chinese herbal medicines on the storage quality of sweet potato

**DOI:** 10.3389/fpls.2025.1623582

**Published:** 2025-07-28

**Authors:** Zengzhi Si, Fengrui Men, Yangyang Liu, Weicao Wang, Jiale Song

**Affiliations:** Hebei Key Laboratory of Crop Stress Biology, Hebei Normal University of Science and Technology, Qinhuangdao, Hebei, China

**Keywords:** sweet potato, storage tolerance, postharvest quality, TOPSIS evaluation, Chinese herbal extracts, optimal concentration

## Abstract

Sweet potato (*Ipomoea batatas* (L.) Lam.) are prone to damage during harvest and postharvest storage, leading to significant economic value depreciation. To mitigate nutritional degradation and improve storability, the application of appropriate preservatives is essential. Conventional preservation techniques, including physical, chemical, and biological approaches —though effective, present inherent limitations. Recent research has prioritized eco-friendly natural preservatives from Chinese herbal medicines as sustainable alternatives to synthetic biocides. In this study, we evaluated the storability of fourteen sweet potato varieties and assessed the preservation of Chinese herbal extracts on varieties with differential storage tolerance by investigating storage quality. The results showed that the purple-fleshed line JK142 (storage-tolerant representative) exhibited the lowest decay rate and moderate dry matter content; the yellow-fleshed cultivar Jishu 25 (moderately storage-tolerant representative) showed intermediate decay rate but the highest dry matter content; the white-fleshed line JK147 (storage-sensitive representative) displayed the highest decay rate and lowest dry matter content. Consequently, lines JK142 (storage-tolerant), Jishu 25 (moderately tolerant), and JK147 (storage-sensitive) were selected as representatives of three distinct storability levels to systematically evaluate the effects of varying concentrations of five Chinese herbal extracts on sweet potato postharvest preservation. The results revealed that the storage-tolerant cultivar JK142 achieved optimal cost-benefit preservation with 2% *Andrographis herba* extract; the moderately tolerant Jishu 25 responded best to 0.5% *Artemisia argyi* extract; the storage-sensitive JK147 required 1% *Andrographis herba* extract. These results substantiate the theoretical framework for developing plant-derived preservatives and highlight cultivar-specific preservation strategies. The study further establishes a foundation for investigating the mechanistic basis of herbal extract efficacy in postharvest management.

## 1 Introduction

Sweet potato (*Ipomoea batatas* (L.) Lam.) is a hexaploid trailing herb belonging to the genus *Ipomoea* in the family Convolvulaceae. Ranked among the world’s staple crops, sweet potato finds its dominant global production base in China, which serves as both the largest producer and consumer while maintaining significant export volumes (Lyu et al., 2021). According to statistics from the Food and Agriculture Organization of the United Nations (FAO), in 2022, China cultivated sweet potatoes on an area of 21.573 million hectares, yielding a total production of 468.3 million metric tons (FAO, 2023). This nutritionally dense crop provides essential phytonutrients like carotenoids and anthocyanins (Ming and Liang, 2020; Nguyen et al., 2021), with clinically demonstrated benefits including anti-aging properties, anticancer potential, blood glucose regulation, and blood pressure modulation (Alam, 2021; Galvao et al., 2021). Furthermore, sweet potatoes constitute a cornerstone crop for global food security. Their balanced nutritional profile, palatability, and high cultivation efficiency render them strategically advantageous for driving rural revitalization while making substantial contributions to food security preservation (Li et al., 2022).

Sweet potatoes exhibit heightened susceptibility to microbiological spoilage and physiological deterioration throughout postharvest handling phases (transportation, storage, and retail), attributed to the interplay of senescence-related biochemical changes, pathological disorders, and colonization by fungal/bacterial pathogens (Wei et al., 2022; Xu et al., 2024). Moreover, sweet potato market dynamics exhibit pronounced seasonality, creating fiscal imbalances between surplus and lean seasons. Consequently, implementing optimized postharvest protocols becomes imperative to enable phased commercialization after bulk harvesting (Li, 2021; He et al., 2023). In China, approximately 30% of sweet potato yields incur postharvest losses stemming from inadequate storage infrastructure, which constitutes a critical impediment to sustainable development across the industry value chain (Pang et al., 2021). Current preservation methods for sweet potatoes encompass a variety of physical, chemical, and biological approaches, including heat treatment, ozone treatment, chemical treatments, coating treatments, and the utilization of microbial cells and their metabolites (Li et al., 2021; Chu et al., 2024). While these methods effectively contribute to sweet potato preservation, each has inherent limitations. Recently, natural extracts have emerged as a prominent research focus for fruit and vegetable preservation.

Numerous traditional Chinese herbal medicines with dual medicinal and edible functions are now recognized as promising green preservatives. Xu et al. demonstrated that licorice extracts, including glycyrrhizic acid (LA) and licorice flavonoids (LF), effectively maintained the appearance and storage quality of fresh-cut sweet potato slices (Xu et al., 2024). Cheng et al. demonstrated that fumigation with 40 μL/L cinnamon essential oil significantly reduced weight loss and preserved higher hardness in sweet potatoes (Cheng et al., 2021). Liu et al. demonstrated that undiluted ginger extract (100% v/v) significantly reduced the incidence of soft rot in sweet potatoes and improved their postharvest quality (Liu et al., 2020). Although common Chinese herbal medicines (e.g., *Andrographis herba*, *Perilla frutescens*, *Artemisia argyi*, *Prunella vulgaris*, and *Schizonepeta tenuifolia*) exhibit antibacterial properties, their efficacy against sweet potato pathogens remains unexplored. This study evaluated the storage tolerance of fourteen sweet potato cultivars and investigated the preservative potential of traditional Chinese herbal extracts across cultivars with differential storage tolerance. This study aims to provide theoretical foundations and technical support for sweet potato postharvest preservation and the development of herbal-based preservatives.

## 2 Results

### 2.1 Comparative analysis of storage tolerance among diverse sweet potato cultivars (lines)

**Figure 1:**
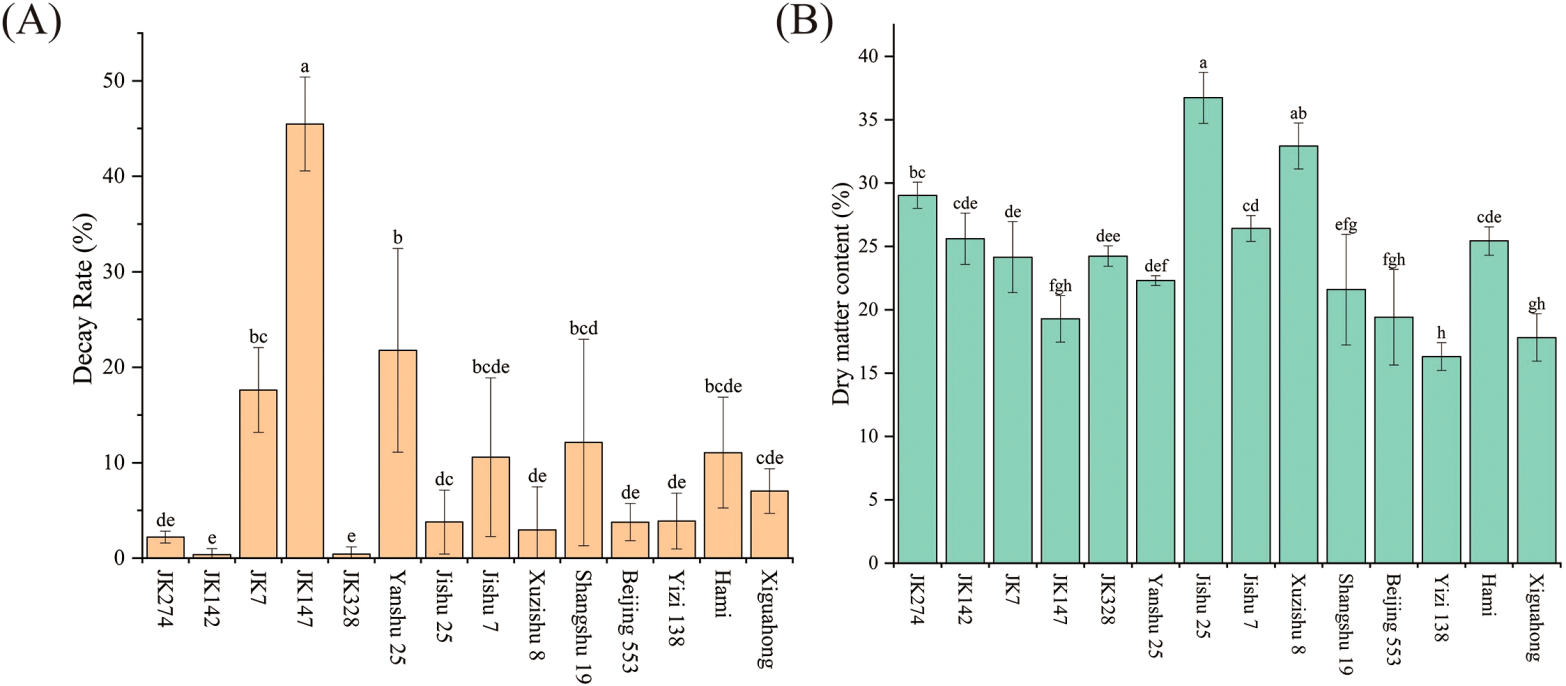
Comparison of storage tolerance among fourteen sweet potato cultivars (lines) after 60 days of storage. **(A)** Decay rate; **(B)** Dry matter content. Error bars and small letters indicate statistical comparisons.

Experimental observations demonstrated that sweet potato cultivars with distinct flesh colors exhibited differential storability, though all varieties displayed progressively increasing decay rates during storage (**Figure 1A**; **Table S1**). After 60 days of storage, lines JK142 and JK328 showed minimal decay (≈0.4%) with no significant difference between them. Lines JK274, Xuzishu 8, Beijing 553, Jishu 25, and Yizi 138 exhibited decay rates of 2-4%—approximately 8 times higher than JK142/JK328, though this difference lacked statistical significance. In contrast, Xiguahong, Jishu 7, Hami, and Shangshu 19 displayed significantly higher decay rates (7-13%, p < 0.05), while lines JK7 and Yanshu 25 showed markedly elevated decay levels (17-22%, p < 0.01). Notably, line JK147 exhibited the highest decay rate (45.48%), measuring 113 times greater than those of JK142 and JK328. After 60 days of storage, Jishu 25 and Xuzishu 8 exhibited dry matter contents exceeding 32%, surpassing other cultivars. Line JK274 demonstrated intermediate dry matter levels (28–32%), while Jishu 7, JK142, Hami, JK328, JK7, Yanshu 25, Shangshu 19, Beijing 553, JK147, Xiguahong, and Yizi 138 all registered dry matter contents below 28% (**Figure 1B**; **Table S1**).

### 2.2 Comparison of storage quality among diverse sweet potato cultivars (lines)

**Figure 2:**
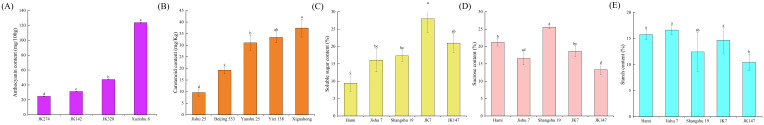
Comparison of storage quality among different sweet potato cultivars (lines) after 60 days of storage. **(A)** Anthocyanin content; **(B)** Carotenoid content; **(C)** Soluble sugar content; **(D)** Sucrose content; **(E)** Starch content. Each chart includes error bars and different lowercase letters above bars indicate statistical significance.

Experimental data demonstrated significant quality divergence among sweet potato cultivars with distinct flesh pigmentation during 60-day storage (**Figure 2**; **Table S2**). Analysis of rotting rates revealed tiered deterioration patterns: JK142 and JK328 maintained optimal storability (0.4% rotting rate), JK274 and Xuzishu 8 showed moderate susceptibility (2-4%), Xiguahong exhibited higher degradation (7-13%), while JK7, Yanshu 25 (17-22%), and JK147 (45.48%) displayed severe physiological decline (**Figure 2A**). Dry matter content stratification occurred across three tiers: Jishu 25 and Xuzishu 8 (>32%) ranked highest, JK274 (28-32%) intermediate, and 11 remaining cultivars (<28%) lower (**Figure 2B**). Purple-fleshed varieties exhibited extreme anthocyanin variation (JK274:24.77 → Xuzishu 8:123.76 mg/100g), whereas yellow-fleshed cultivars showed progressive carotenoid accumulation (Jishu 25:9.58 → Xiguahong:37.42 mg/kg) (**Figures 2C, D**). Starch-type cultivars exhibited marked soluble sugar disparities (Hami 9.45% ↔ JK7 28.00%), with sucrose levels peaking in Shangshu 19 (25.53%) and starch content maximized in Jishu 7 (16.63%) (**Figure 2E**). All parameters displayed statistically significant cultivar-specific patterns (p<0.05), confirming flesh coloration as a critical determinant of postharvest performance.

### 2.3 Cluster analysis of diverse sweet potato cultivars (lines)

**Figure 3:**
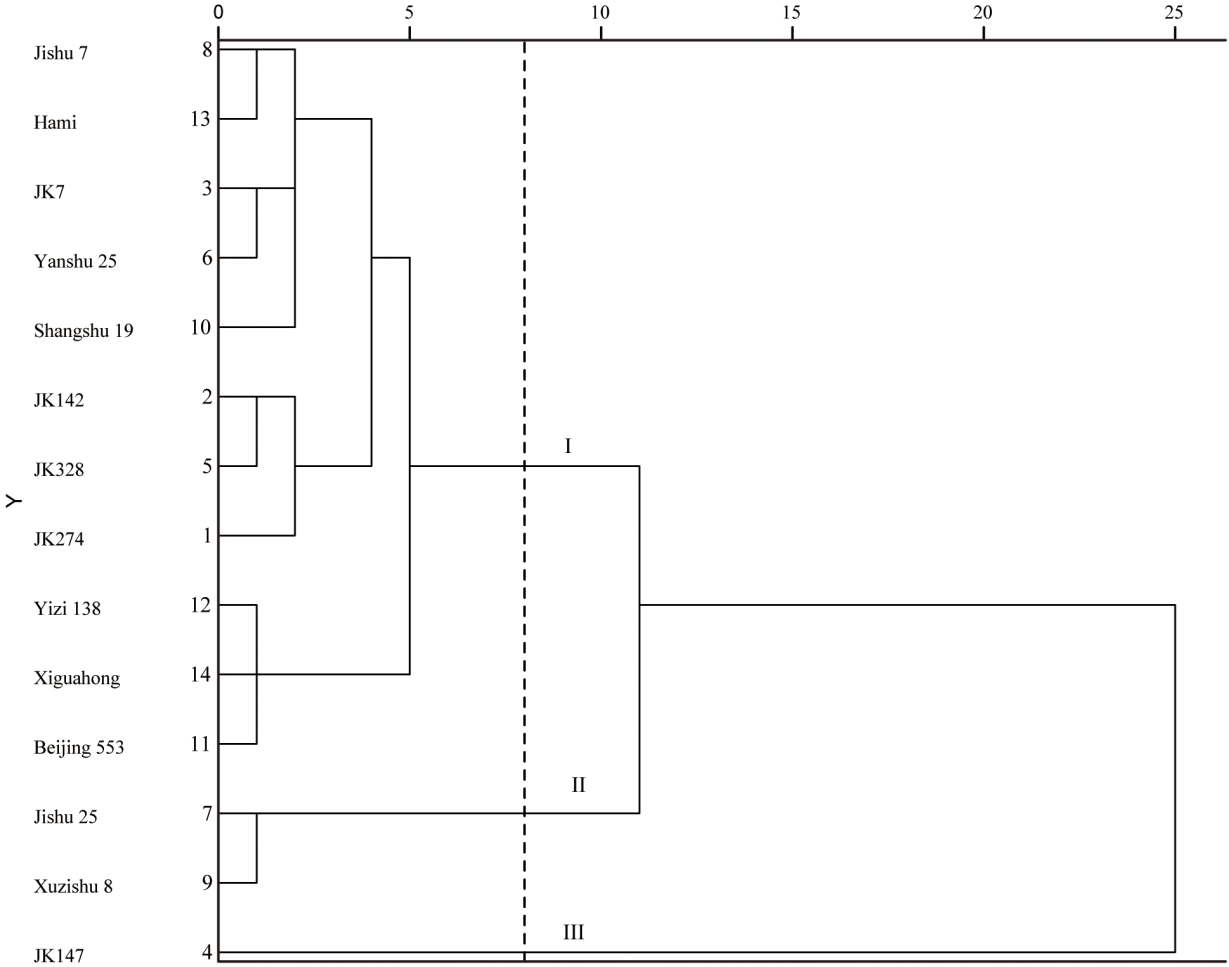
Cluster analysis based on storage performance over 60 days for fourteen sweet potato cultivars (lines).

Cluster analysis using squared Euclidean distance classified 14 sweet potato cultivars (lines) into three distinct groups based on standardized rotting rates and dry matter content (**Figure 3**; **Table S3**). At a squared Euclidean distance threshold of 8, the cultivars segregated as follows: Group I comprised 11 materials (Jishu 7, Hami, JK7, Yanshu 25, Shangshu 19, JK142, JK328, JK274, Yizi 138, Xiguahong, Beijing 553) exhibiting moderate rotting rates and intermediate dry matter content (28-32%), collectively demonstrating enhanced storability, with the purple-fleshed line JK142 showing the highest storage resilience. Group II included two cultivars (Jishu 25, Xuzishu 8) characterized by elevated dry matter content (>32%) and moderate rotting rates (2-4%), representing intermediate storability, of which Jishu 25 displayed the maximal dry matter accumulation. Group III consisted solely of line JK147, registering the highest rotting rate (45.48%) combined with the lowest dry matter content (<26%), confirming its extreme susceptibility to postharvest deterioration.

### 2.4 Effects of five traditional Chinese herbal medicines on the storage and preservation of sweet potato cultivar JK142

**Figure 4:**
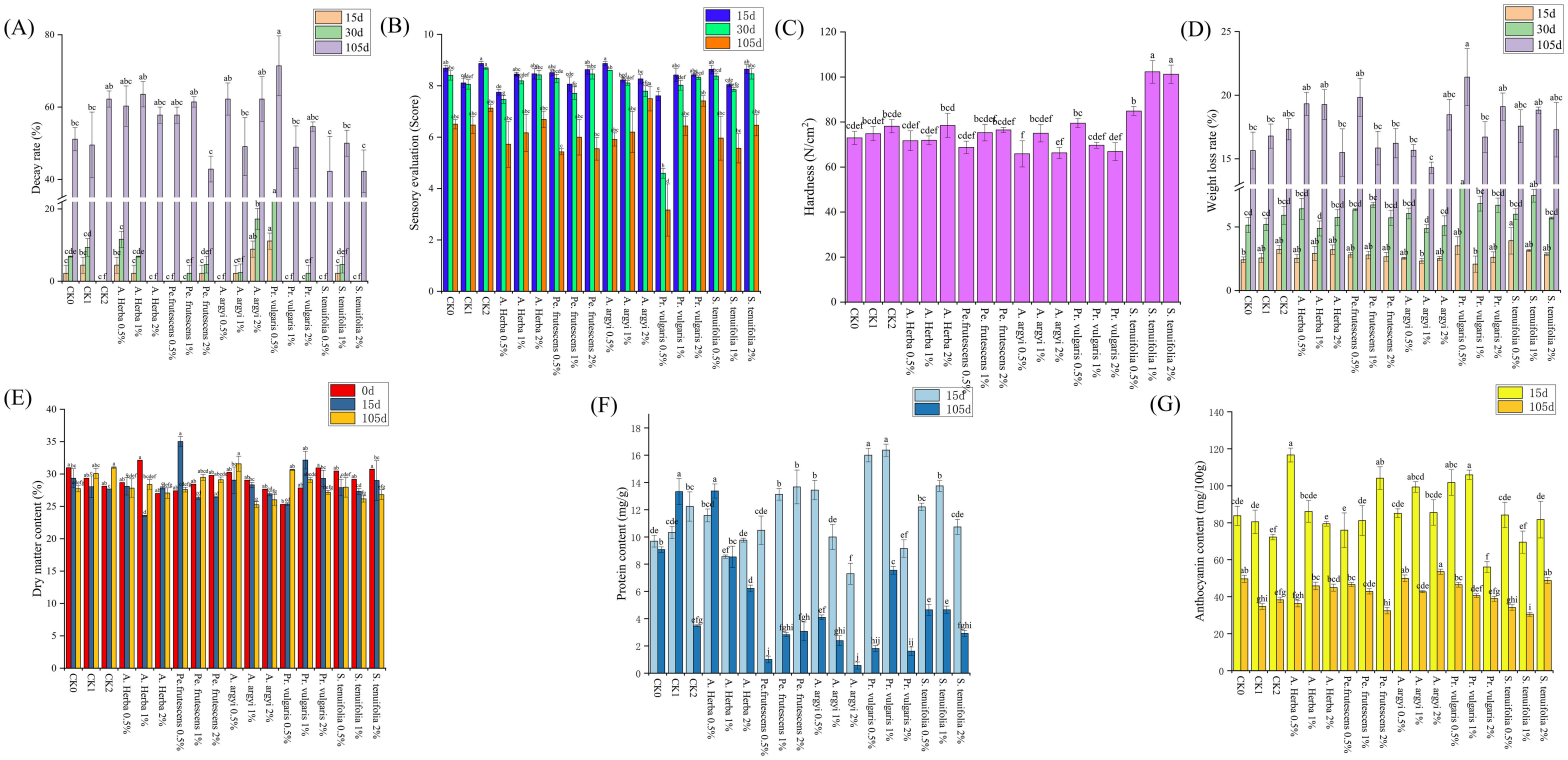
Effects of different extraction concentrations of five Chinese herbal medicines on the variety JK142 during different storage periods. **(A)** Decay rate; **(B)** Sensory evaluation; **(C)** Hardness; **(D)** Weight loss rate; **(E)** Dry matter content; **(F)**Protein content; **(G)** Anthocyanin content. Different lowercase letters above bars indicate statistical significance.

The decay rate of sweet potato cultivar JK142 showed significant variations across different storage periods and concentrations of Chinese herbal treatments (**Figure 4**; **Table S4**). At 15 days of storage, the 2% *Artemisia argyi* treatment exhibited markedly higher decay rates (8.89%) compared to the control group, representing the highest decay percentage among all treatments. By day 30, most treatments showed progressively increasing decay rates with varying escalation patterns, while the 2% *Andrographis herba*, 0.5% *Perilla frutescens*, 0.5% *Artemisia argyi*, 1% *Prunella vulgaris*, 0.5% *Schizonepeta tenuifolia*, and 2% *Schizonepeta tenuifolia* groups maintained stable decay rates. At the final observation (105 days), the lowest decay rates were recorded in both 0.5% and 2% *Schizonepeta tenuifolia* treatments, demonstrating the preservative efficacy of this herbal treatment for JK142 storage (**Figure 4A**).

After one month of storage, the sensory attributes of JK142 sweet potatoes showed gradual improvement with increasing concentrations of *Andrographis herba* and *Prunella vulgaris*. In contrast, treatments with *Perilla frutescens* and *Schizonepeta tenuifolia* initially reduced sensory quality before showing improvement at higher concentrations. Treatment with *Artemisia argyi* consistently decreased quality throughout the concentration range. After 105 days of storage, significant deterioration in overall sensory quality was observed, along with increased decay rates (**Figure 4B**). Regarding texture, hardness values of JK142 showed minimal variation under *Andrographis herba*, *Perilla frutescens*, *Artemisia argyi*, and *Prunella vulgaris* treatments, with no statistically significant differences. However, *Schizonepeta tenuifolia*-treated sweet potatoes maintained consistently higher hardness, peaking at 1% concentration (**Figure 4C**).

Initially, no significant differences were observed in weight loss rates across treatments. After 30 days of storage, however, 1% concentrations of *Andrographis herba* and *Artemisia argyi* significantly reduced weight loss in JK142 sweet potatoes. Throughout storage, 1% *Artemisia argyi* proved particularly effective at minimizing both water loss and nutrient depletion (**Figure 4D**). Dry matter content exhibited dynamic fluctuations during storage, generally declining over time. Notably, treatments with 0.5% *Andrographis herba*, 0.5% *Artemisia argyi*, 0.5% *Prunella vulgaris*, 2% *Andrographis herba*, 1% *Prunella vulgaris*, and 1% *Perilla frutescens* all effectively slowed dry matter loss (**Figure 4E**).

Compared to other herbal treatments, 1% *Prunella vulgaris* effectively reduced protein loss in sweet potato tubers during early storage. Protein content showed a general decline across all treatments over time, though 0.5% *Andrographis herba* maintained protein levels most effectively by day 105 (**Figure 4F**). Anthocyanin content under 0.5% *Andrographis herba* treatment was 1.4-fold higher than controls at day 15. By day 105, all treatments showed anthocyanin reduction, with 2% *Artemisia argyi* most effectively preserving anthocyanin content in JK142 (**Figure 4G**).

In our study, data processing and analysis were conducted using the Scientific Platform Serving for Statistics Professionals (SPSSPRO) to evaluate storage effectiveness rankings of sweet potatoes across different treatment groups. The results, presented in **Table 1**, indicate the order of effectiveness from highest to lowest as follows: CK > 2% *Andrographis herba* > CK_1_ > 1% *Prunella vulgaris* > 1% *Andrographis herba* > 1% *Artemisia argyi* > 2% *Schizonepeta tenuifolia* > 0.5% *Andrographis herba* > 2% *Artemisia argyi* > 0.5% *Artemisia argyi* > 0.5% *Schizonepeta tenuifolia* > 1% *Perilla frutescens* > 0.5% *Perilla frutescens* > 0.5% *Prunella vulgaris* > 2% *Perilla frutescens* > CK_2_ > 1% *Schizonepeta tenuifolia* > 2% *Prunella vulgaris*.

**Table 1:** TOPSIS evaluation of storage effectiveness for line JK142.

Processing level	Positive ideal solution distance(D^+^)	Negative ideal solution distance(D^-^)	Comprehensive score index	Sort
Blanks CK	0.255	0.761	0.749	1
Clear Water CK_1_	0.488	0.709	0.592	3
Chemistry CK_2_	0.651	0.416	0.39	16
*Andrographis herba* 0.5%	0.601	0.62	0.508	8
*Andrographis herba* 1%	0.481	0.567	0.541	5
*Andrographis herba* 2%	0.412	0.662	0.616	2
*Perilla frutescens* 0.5%	0.677	0.471	0.41	13
*Perilla frutescens* 1%	0.623	0.46	0.425	12
*Perilla frutescens* 2%	0.746	0.479	0.391	15
*Artemisia argyi* 0.5%	0.615	0.527	0.462	10
*Artemisia argyi* 1%	0.544	0.629	0.536	6
*Artemisia argyi* 2%	0.67	0.591	0.469	9
*Prunella vulgaris* 0.5%	0.776	0.515	0.399	14
*Prunella vulgaris* 1%	0.453	0.578	0.561	4
*Prunella vulgaris* 2%	0.721	0.401	0.358	18
*Schizonepeta tenuifolia* 0.5%	0.628	0.503	0.445	11
*Schizonepeta tenuifolia* 1%	0.682	0.412	0.376	17
*Schizonepeta tenuifolia* 2%	0.537	0.62	0.536	7

### 2.5 Effects of five traditional Chinese herbal medicines on the storage and preservation of sweet potato cultivar Jishu 25

**Figure 5:**
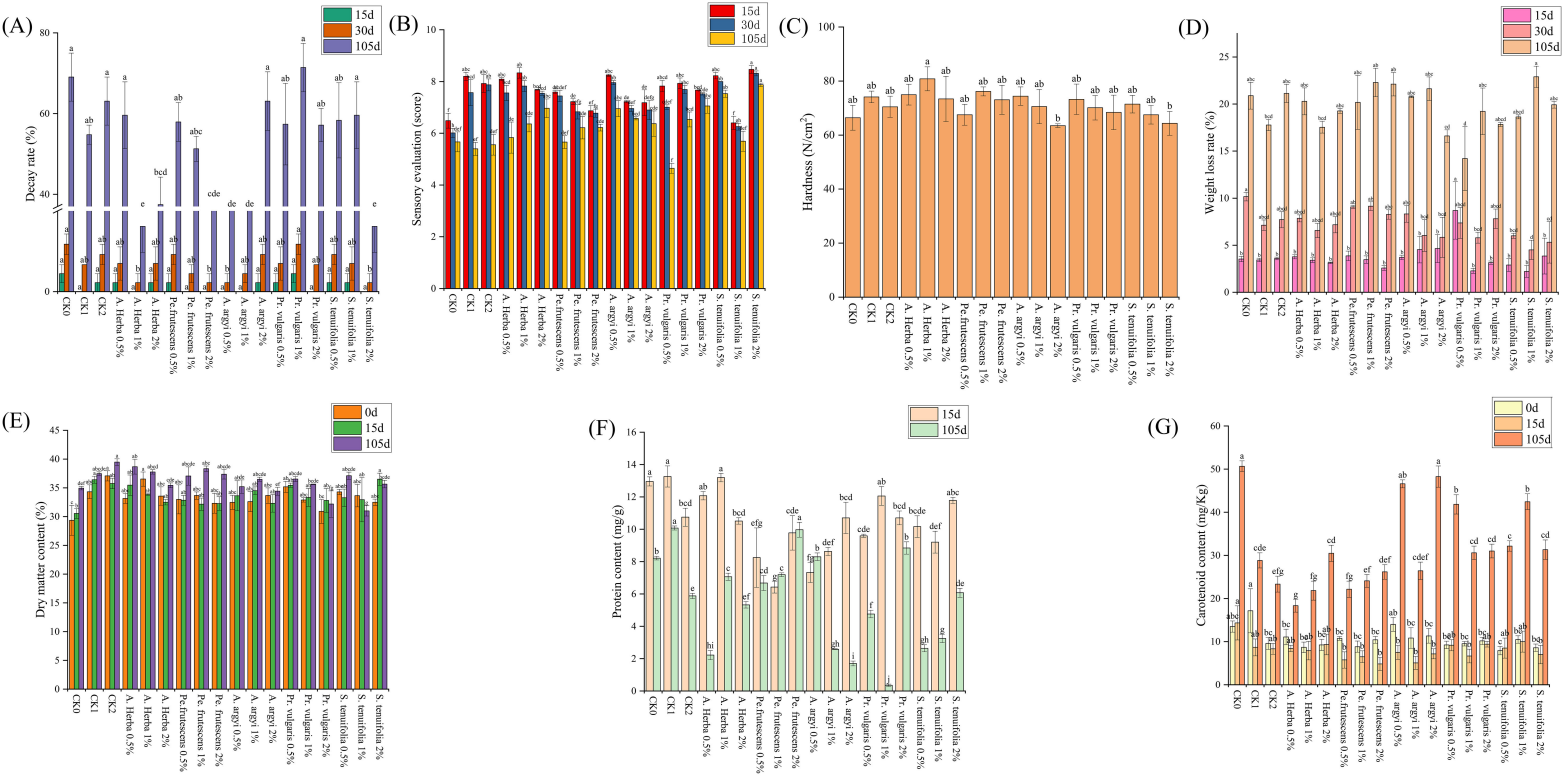
Effects of different extraction concentrations of five Chinese herbal medicines on Jishu 25 during different storage periods. **(A)** Decay rate; **(B)** Sensory evaluation; **(C)** Hardness; **(D)** Weight loss rate; **(E)** Dry matter content; **(F)**Protein content; **(G)** Carotenoid content. Different lowercase letters above bars indicate statistical significance.

As shown in **Figure 5**, Jishu 25 potatoes showed no decay after 15 days when treated with: 1%*Andrographis herba*, 1% and 2% *Perilla frutescens*, 0.5% and 1% *Artemisia argyi*, 2% *Prunella vulgaris*, and 2% *Schizonepeta tenuifolia* (**Figure 5**; **Table S5**). Throughout storage, decay rates remained low with 1% *Andrographis herba*, 0.5% *Artemisia argyi*, and 2% *Schizonepeta tenuifolia* treatments (**Figure 5A**). Sensory quality declined across all herbal treatments over time, with particularly severe deterioration in 0.5% *Prunella vulgaris*-treated potatoes during later stages (**Figure 5B**). Hardness initially increased then decreased with rising *Andrographis herba* and *Perilla frutescens* concentrations, while *Artemisia argyi*, *Prunella vulgaris*, and *Schizonepeta tenuifolia* treatments showed progressive softening. Notably, 1% *Andrographis herba* and 1% *Perilla frutescens* maintained consistently high hardness throughout storage (**Figure 5C**).

During early-to-mid storage, 1% *Prunella vulgaris* and 1% *Schizonepeta tenuifolia* treatments effectively reduced weight loss in Jishu 25. By day 105, 0.5% *Prunella vulgaris* best preserved moisture and nutrient content. Weight loss changes remained minimal with 1% *Andrographis herba* and 2% *Artemisia argyi* treatments throughout storage (**Figure 5D**). *Schizonepeta tenuifolia* at high concentrations maintained elevated dry matter content initially, while 0.5% *Andrographis herba* showed highest dry matter retention by day 105. Overall, 0.5% *Andrographis herba*, 1% *Perilla frutescens*, and 0.5% *Schizonepeta tenuifolia* all significantly preserved dry matter content during extended storage (**Figure 5E**).

Protein content declined across all Jishu 25 treatments except for 1-2% *Perilla frutescens* and 0.5% *Artemisia argyi*. Notably, 0.5% *Perilla frutescens* and 2% *Prunella vulgaris* treatments effectively mitigated protein loss during storage (**Figure 5F**). Carotenoid content was initially highest with 0.5% *Artemisia argyi* treatment. After 15 days, 1% *Schizonepeta tenuifolia* showed better performance. By day 105, carotenoid content increased significantly, with superior results from: 2% *Andrographis herba*, 2% *Perilla frutescens*, 2% *Artemisia argyi*, 0.5% *Prunella vulgaris*, and 1% *Schizonepeta tenuifolia* treatments (**Figure 5G**).

Subsequently, we processed and analyzed data using SPSSPRO to evaluate storage effectiveness rankings for sweet potatoes across different treatment groups. As shown in **Table 2**, the treatments were ranked by effectiveness in descending order as follows: 0.5% *Artemisia argyi* > 2% *Schizonepeta tenuifolia* > 1% *Andrographis herba* > 0.5% *Prunella vulgaris* > 2% *Andrographis herba* > 2% *Perilla frutescens* > CK_1_ > 2% *Artemisia argyi* > 2% *Prunella vulgaris* > CK > 1% *Artemisia argyi* > 0.5% *Schizonepeta tenuifolia* > 1% *Perilla frutescens* > 0.5% *Perilla frutescens* > CK_2_ > 1% *Schizonepeta tenuifolia* > 1% *Prunella vulgaris* > 0.5% *Andrographis herba*.

**Table 2:** TOPSIS evaluation of storage effectiveness for Jishu 25.

Processing level	Positive ideal solution distance(D^+^)	Negative ideal solution distance(D^-^)	Comprehensive score index	Sort
Blanks CK	0.654	0.586	0.473	10
Clear Water CK_1_	0.565	0.565	0.5	7
Chemistry CK_2_	0.731	0.409	0.359	15
*Andrographis herba* 0.5%	0.772	0.354	0.314	18
*Andrographis herba* 1%	0.484	0.698	0.59	3
*Andrographis herba* 2%	0.498	0.528	0.515	5
*Perilla frutescens* 0.5%	0.687	0.397	0.366	14
*Perilla frutescens* 1%	0.672	0.457	0.405	13
*Perilla frutescens* 2%	0.583	0.591	0.504	6
*Artemisia argyi* 0.5%	0.391	0.746	0.657	1
*Artemisia argyi* 1%	0.614	0.543	0.469	11
*Artemisia argyi* 2%	0.603	0.576	0.489	8
*Prunella vulgaris* 0.5%	0.567	0.622	0.523	4
*Prunella vulgaris* 1%	0.762	0.36	0.321	17
*Prunella vulgaris* 2%	0.571	0.538	0.485	9
*Schizonepeta tenuifolia* 0.5%	0.599	0.492	0.451	12
*Schizonepeta tenuifolia* 1%	0.759	0.388	0.338	16
*Schizonepeta tenuifolia* 2%	0.448	0.705	0.611	2

### 2.6 Effects of five traditional Chinese herbal medicines on the storage and preservation of sweet potato cultivar JK147

**Figure 6:**
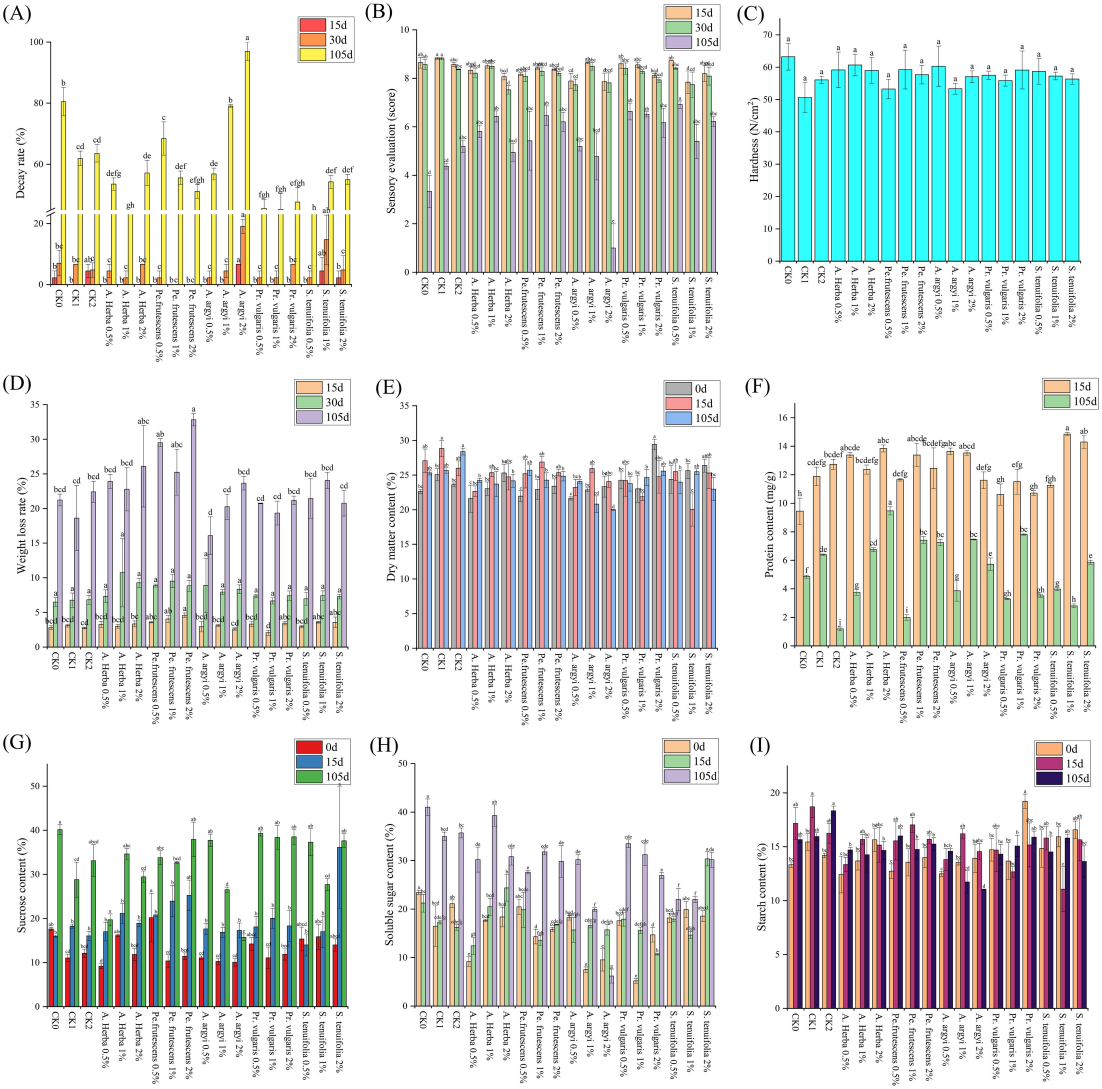
Effects of different extraction concentrations of five Chinese herbal medicines on the variety JK147 at different storage periods. **(A)** Decay rate; **(B)** Sensory evaluation; **(C)** Hardness; **(D)** Weight loss rate; **(E)** Dry matter content; **(F)** Protein content; **(G)** Sucrose content; **(H)** Soluble sugar content; **(I)** Starch content. Different lowercase letters above bars indicate statistical significance.

**Figure 6** demonstrates that the decay rate of sweet potato variety JK147 increases with extended storage duration (**Figure 6**; **Table S6**). During mid-storage, 1-2% *Perilla frutescens* treatments effectively reduced decay incidence. Overall, 2% *Artemisia argyi* promoted decay, while 1% *Andrographis herba* and 0.5% concentrations of *Artemisia argyi*, *Prunella vulgaris*, and *Schizonepeta tenuifolia* better preserved freshness (**Figure 6A**). In late storage, sensory quality declined markedly - 2% *Artemisia argyi*-treated potatoes showed near-total decay. In contrast, JK147 treated with 0.5% *Schizonepeta tenuifolia* achieved the highest sensory scores, differing significantly from controls (**Figure 6B**).

Under *Schizonepeta tenuifolia* treatment, JK147 hardness gradually decreased, while 1% *Andrographis herba* and 1% *Perilla frutescens* treatments maintained consistently high hardness levels (**Figure 6C**). The 1% *Prunella vulgaris* treatment showed optimal performance during early and mid-storage of JK147, demonstrating the lowest weight loss rate. In late storage, low-concentration *Artemisia argyi* exhibited minimal weight loss variation with the lowest rate among all treatments (**Figure 6D**). Significant variations in dry matter content occurred across herbal treatments, with 2% *Andrographis herba*, 0.5% *Prunella vulgaris*, and 1% *Schizonepeta tenuifolia* most effectively preserving JK147 dry matter content (**Figure 6E**). Protein content declined variably with storage duration, though 2% *Andrographis herba* and 1% *Prunella vulgaris* treatments proved most effective at protein preservation and loss reduction (**Figure 6F**).

After 105 days of storage, treatments containing 1% *Andrographis herba*, 2% *Perilla frutescens*, 0.5% *Artemisia argyi*, 0.5% *Prunella vulgaris*, and 2% *Schizonepeta tenuifolia* showed the highest sucrose content (**Figure 6G**). During initial 15-day storage, 2% *Andrographis herba* and 2% *Schizonepeta tenuifolia* treatments yielded higher soluble sugar content than controls. By day 105, 1% *Andrographis herba* treatment maintained superior soluble sugar content compared to both water and untreated controls, demonstrating its effectiveness in reducing late-stage sugar loss (**Figure 6H**). Starch content analysis revealed that 1% *Perilla frutescens* showed initial superiority, while 0.5% *Perilla frutescens* performed better in later stages. Extended storage demonstrated that 0.5% *Prunella vulgaris*, 1% *Schizonepeta tenuifolia*, and 2% *Andrographis herba* treatments most effectively preserved JK147 starch content (**Figure 6I**).

In our study, data processing and analysis were performed using SPSSPRO to evaluate the storage effectiveness ranking of sweet potatoes across different treatment groups. As shown in **Table 3**, the treatments are ranked by effectiveness in descending order as follows: 1% *Prunella vulgaris* > 1% *Andrographis herba* > CK_1_ > 1% *Perilla frutescens* > 2% *Andrographis herba* > 2% *Prunella vulgaris* > 2% *Perilla frutescens* > 2% *Schizonepeta tenuifolia* > 0.5% *Artemisia argyi* > 0.5% *Prunella vulgaris* > CK > 0.5% *Schizonepeta tenuifolia* > CK_2_ > 1% *Schizonepeta tenuifolia* > 0.5% *Andrographis herba* > 0.5% *Perilla frutescens* > 1% *Artemisia argyi* > 2% *Artemisia argyi*.

**Table 3:** TOPSIS evaluation of storage effectiveness for line JK147.

Processing level	Positive ideal solution distance(D^+^)	Negative ideal solution distance(D^-^)	Comprehensive score index	Sort
Blanks CK	0.444	0.666	0.600	11
Clear Water CK_1_	0.347	0.674	0.660	3
Chemistry CK_2_	0.508	0.723	0.587	13
*Andrographis herba* 0.5%	0.541	0.540	0.499	15
*Andrographis herba* 1%	0.360	0.721	0.667	2
*Andrographis herba* 2%	0.398	0.685	0.632	5
*Perilla frutescens* 0.5%	0.567	0.558	0.496	16
*Perilla frutescens* 1%	0.370	0.673	0.645	4
*Perilla frutescens* 2%	0.437	0.697	0.615	7
*Artemisia argyi* 0.5%	0.435	0.667	0.605	9
*Artemisia argyi* 1%	0.609	0.519	0.460	17
*Artemisia argyi* 2%	0.865	0.308	0.263	18
*Prunella vulgaris* 0.5%	0.456	0.688	0.601	10
*Prunella vulgaris* 1%	0.273	0.782	0.742	1
*Prunella vulgaris* 2%	0.406	0.695	0.631	6
*Schizonepeta tenuifolia* 0.5%	0.451	0.672	0.599	12
*Schizonepeta tenuifolia* 1%	0.512	0.560	0.523	14
*Schizonepeta tenuifolia* 2%	0.419	0.658	0.611	8

## 3 Discussion

This study investigated storage tolerance indices and quality indicators across 14 sweet potato cultivars (lines). Through statistical analyses - including general linear modeling, cluster analysis, and correlation analysis - we systematically examined interrelationships among these parameters. Results identified JK142, Jishu 25, and JK147 as representative cultivars exhibiting three distinct levels of storage tolerance. These findings provide valuable guidance for sweet potato variety selection in storage applications, offering potential improvements for shelf-life extension and quality maintenance in agricultural and food industry practices.

In sweet potato storage, tolerance is evaluated through indicators including decay rate, sensory quality, hardness, and weight loss rate (Wang et al., 2016), while freshness quality is assessed via protein and dry matter content (Wei, 2021; Jiang, 2024). This study examined the preservation effects of traditional Chinese medicinal herbs on these parameters. The JK142 line treated with high concentrations of *Schizonepeta tenuifolia* demonstrated superior performance during both short-term (0–30 days) and long-term (30–105 days) storage, showing reduced decay and weight loss rates alongside better maintained sensory quality and hardness. These results suggest *Schizonepeta tenuifolia* effectively preserves water and nutrient retention in JK142, thereby enhancing marketability (Ai et al., 2013). Notably, purple sweet potatoes’ anthocyanin content contributes significant antioxidant and free radical scavenging properties (Grace et al., 2014; Grace et al., 2015). Our findings indicate that 1% *Prunella vulgaris* and 1% *Andrographis herba* are optimal for short-term and long-term JK142 storage respectively, effectively preserving dry matter content while minimizing protein and anthocyanin loss. Through TOPSIS analysis incorporating storage tolerance, nutritional quality, and cost-effectiveness metrics, we determined that 2% *Andrographis herba* provides the most comprehensive benefits for storing the tolerant JK142 line.

Treatment of the sweet potato variety Jishu 25 with 2% *Schizonepeta tenuifolia* extract demonstrated significant preservation effects, maintaining low rot incidence while preserving weight, sensory quality, and hardness throughout storage. This cultivar’s notable carotenoid content, associated with reduced risks of cardiovascular diseases, night blindness, and cancer (Adu et al., 2025), was effectively preserved under different treatment conditions. Short-term storage analysis revealed that 2% *Schizonepeta tenuifolia* extract enhanced dry matter and protein retention, while the 1% concentration better preserved carotenoids. For long-term storage, 2% *Perilla frutescens* extract proved most effective in maintaining all measured nutritional components (dry matter, protein, and carotenoids). The TOPSIS multi-criteria evaluation, incorporating storage tolerance, nutritional quality, and cost parameters, identified 0.5% *Artemisia argyi* extract as optimal for preserving the moderately tolerant JK142 line.

During short-term storage, treatment of sweet potato variety JK147 with 0.5% *Artemisia argyi* extract demonstrated multiple beneficial effects: reduced rot incidence, enhanced firmness, improved sensory quality, and decreased weight loss rate. Simultaneously, 2% *Prunella vulgaris* extract application effectively maintained elevated levels of key nutritional components including dry matter, protein, sucrose, soluble sugars, and starch. For long-term preservation, both 0.5% *Schizonepeta tenuifolia* and 2% purple perilla (*Perilla frutescens*) extracts showed significant quality-preserving effects on JK147. Through comprehensive TOPSIS evaluation incorporating storage tolerance metrics, nutritional quality parameters, and cost-benefit analysis, 1% *Andrographis herba* extract was identified as an optimal green preservative solution for this non-storage-tolerant sweet potato variety.

## 4 Materials and methods

### 4.1 Plant materials

This study utilized five proprietary sweet potato cultivars from our laboratory collection: Jikezi 18 (JK274), Jikezi 19 (JK142), Jikeshu 20 (JK7), Jikeshu 21 (JK147), and Jikezi 22 (JK328). The experimental materials also included nine commercially grown varieties representative of eastern Hebei’s sweet potato production: Yanshu 25, Jishu 25, Jishu 7, Xuzishu 8, Shangshu 19, Beijing 553, Yizi 138, Hami, and Xiguahong.

### 4.2 Comparison of storage tolerance among different sweet potato cultivars (lines)

The experiment employed three biological replicates per cultivar, each containing approximately 100 sweet potatoes. Tubers were packed in 40 cm × 70 cm polypropylene woven bags and stored for 60 days under controlled conditions. Post-storage evaluations included quantification of decay rate and dry matter content for each cultivar/line (Wang XJ., 2016; Wang ZY., 2016). Cluster analysis was performed to classify the 14 cultivars/lines based on their storage tolerance characteristics. Additionally, Pearson correlation analysis between decay rate and dry matter content provided preliminary insights into storage quality parameters.

### 4.3 Comparison of storage quality among different sweet potato cultivars (lines)

#### 4.3.1 Comparison of anthocyanin content

Following 60-day storage, we evaluated the anthocyanin content across four purple-fleshed sweet potato cultivars/lines (JK274, JK142, JK328, and Xuzishu 8). The experimental design employed three biological replicates per treatment, with each replicate representing an individual cultivar. Subsequent analysis examined both the absolute anthocyanin levels and their correlation with respective storage performance metrics (Yan et al., 2022).

#### 4.3.2 Comparison of carotenoid content

The carotenoid content of five fresh-eating sweet potato varieties was compared after 60 days of storage. Each variety was a single replicate, with three replicates per treatment. The carotenoid content of Yanshu 25, Jishu 25, Beijing 553, Yizi 138, and Xiguahong was analyzed (Li et al., 2016), and its correlation with the corresponding storage level was assessed.

#### 4.3.3 Comparison of sugar and starch content

After 60 days of storage, the sugar and starch content of five starch-type sweet potato cultivars (lines) was compared. Each cultivar was a single replicate, with three replicates per treatment. The soluble sugar and sucrose content of JK7, JK147, Jishu No. 7, Shangshu 19, and Hami were determined (Pei, 2022; Wu et al., 2023). The starch content was calculated based on the relationship between starch and dry matter content (Wang et al., 1989; Yang et al., 2023). The correlations between starch content, dry matter content, and decay rate were analyzed separately.

### 4.4 Storage and preservation of the storage-tolerant sweet potato line JK142

The experimental design evaluated five traditional Chinese medicinal herb extracts at three concentrations (0.5%, 1%, and 2%), with CK serving as the blank control and CK1 as the aqueous control. A chemical control (CK2) using 500 mg/kg carbendazim was included. Treatment solutions were prepared by soaking herbs/chemicals in water for 2 hours, followed by tuber immersion for 30 minutes and subsequent air-drying. Each treatment group comprised 90 sweet potato tubers for primary evaluation, supplemented with 15 additional tubers for specific storage tolerance assessments. The experiment employed a triplicate design. Storage conditions maintained 12 ± 5°C and 70 ± 10% relative humidity for 105 days. Pre-storage measurements included tuber hardness (Wang ZY., 2016). Periodic evaluations at 0, 15, 30, and 105 days assessed decay rate, weight loss percentage, and sensory characteristics (Wang XJ., 2016). Anthocyanin and protein content were quantified at 15 and 105 days (Wang, 2012; Xiao, 2019), while dry matter content was determined at 0, 15, and 105 days.

### 4.5 Storage and preservation of the moderately storage-tolerant sweet potato cultivar Jishu 25

The study utilized Jishu 25, a widely cultivated light yellow-fleshed sweet potato variety from eastern Hebei Province, selected for its moderate storage tolerance. Carotenoid content was quantified at three storage intervals (0, 15, and 105 days), while all other experimental procedures adhered to the methodology outlined in Section 4.4.

### 4.6 Storage and preservation of the non-storage-tolerant sweet potato line JK147

We selected JK147, a distinctive white-fleshed sweet potato line developed in our laboratory, characterized by low storage tolerance, for this investigation. Sucrose, soluble sugar, and starch contents were analyzed at three storage intervals (0, 15, and 105 days), while all other experimental protocols followed the methodology detailed in Section 4.4.

## 5 Conclusions

In a comprehensive screening study evaluating 14 sweet potato cultivars with differential storage tolerance, three distinct groups emerged based on 60-day storage performance metrics including decay rates and dry matter content. The purple-fleshed JK142 line demonstrated superior storage tolerance, while yellow-fleshed Jishu 25 exhibited moderate tolerance, and white-fleshed JK147 showed limited storage capability. These varieties were strategically selected as model systems due to their characteristic nutritional profiles: JK142’s anthocyanin richness, Jishu 25’s carotenoid content, and JK147’s carbohydrate composition (sucrose, soluble sugars, and starch). Subsequent investigations established optimal herbal treatment protocols: 2% *Andrographis herba* for storage-tolerant JK142, 0.5% *Artemisia argyi* for moderately tolerant Jishu 25, and 1% *Andrographis herba* for non-tolerant JK147. Herbal extracts present significant advantages over synthetic preservatives, offering safer, eco-friendly alternatives with broad-spectrum antimicrobial activity, though their mechanisms in sweet potato preservation require deeper investigation. While current findings validate their preservation efficacy, further research is needed to elucidate impacts on nutritional quality and antioxidant properties during storage. This research direction supports the development of natural preservative systems and standardized industrial applications, aligning with global trends toward sustainable food preservation technologies.

## Data Availability

The original contributions presented in the study are included in the article/**Supplementary Material**. Further inquiries can be directed to the corresponding author.
